# Bio-Based Crosslinked Polymers Synthesized from Functionalized Soybean Oil and Squalene by Thiol–Ene UV Curing

**DOI:** 10.3390/ma14102675

**Published:** 2021-05-20

**Authors:** Sigita Grauzeliene, Deimante Valaityte, Greta Motiekaityte, Jolita Ostrauskaite

**Affiliations:** Department of Polymer Chemistry and Technology, Kaunas University of Technology, Radvilenu Rd. 19, 50254 Kaunas, Lithuania; sigita.grauzeliene@ktu.lt (S.G.); deimante.valaityte@ktu.edu (D.V.); greta.motiekaityte@ktu.edu (G.M.)

**Keywords:** thiol–ene, click reaction, soybean oil, hexathiolated squalene, UV curing, bio-based crosslinked polymer

## Abstract

The development of polymers photopolymerized from renewable resources are extensively growing as fulfills green chemistry and green engineering principles. With the rapid growth of consumerism, research on innovative starting materials for the preparation of polymers may help to reduce the negative impact of petroleum-based plastic materials on the global ecosystem and on animal and human health. Therefore, bio-based crosslinked polymers have been synthesized from functionalized soybean oil and squalene by thiol–ene ultra-violet (UV) curing. First, thiol–ene UV curing of squalene was performed to introduce thiol functional groups. Then, hexathiolated squalene was used as a crosslinker in click UV curing of acrylated epoxidized soybean oil. Two photoinitiators, 2-hydroxy-2-methylpropiophenone and ethylphenyl (2,4,6-trimethylbenzoyl) phosphinate, were tested in different quantities. Rheological properties of the resins were monitored by real-time photorheometry. The characterization of obtained polymers was performed by differential scanning calorimetry, thermogravimetry, and Shore A hardness measurements. Polymers possessed higher storage modulus, thermal characteristics, Shore A hardness, and lower swelling value when ethylphenyl (2,4,6-trimethylbenzoyl) phosphinate was used as photoinitiator.

## 1. Introduction

The main idea of green chemistry and chemical engineering is to reduce the negative impact on environmental and human health caused by the chemical industry [1]. The principles of green chemistry are based on safer chemical design and synthesis, the use of safer solvents and renewable raw materials, as well as real-time analysis to avoid the formation of hazardous substances [2]. These principles can be implemented by designing the structure and synthesis of the products, planning the research methods and processing after the use. Most of the polymers produced today are non-biodegradable petroleum-based polymers that generate large amounts of plastic waste, which has a significant impact on human health and the environment [3]. Thus, in view of sustainable development, the reduction of petroleum-based origin and the use of hazardous materials, new paths are being sought towards more eco-friendly polymers.

Polymers synthesized from renewable raw materials are already used in bio-medical and hygiene products, other consumer goods, agriculture, electronics, packaging, cosmetics, and even tried in optical 3D printing [4,5,6,7,8]. Vegetable oils are an attractive renewable resource as they are cheap, abundant, and easily modifiable [9]. Acrylated epoxidized soybean oil (AESO) is already used in industry and has a trademark Ebecryl 860 [10]. AESO was copolymerized with various plant-derived acrylates obtained from vanillin, plant extractives, hemicellulose, other vegetable oils to give polymers with high biorenewable carbon content of 75–82% [8,11,12]. AESO has also been used in the thiol–ene UV curing reactions with various petroleum-based thiols [13,14], as thiol–ene reaction is one of the most effective polymerization methods of AESO acrylic groups. In this reaction, not only thiol–ene step-growth polymerization, but also chain-growth homopolymerization of AESO can occur [15,16]. AESO has already been used for thiol–ene/thiol-epoxy dual curing with petroleum-based thiols and resulted in thermosets with higher rigidity compared to UV-cured thiol–ene polymers [17].

Some bio-based thiols have been used instead of petroleum-based thiols in the synthesis of polymers. Wang et al. reported thiolated oligomer synthesized by esterification of castor oil with 3-mercaptopropionic acid [18]. The polymerization of thiolated oligomer was initiated under UV irradiation without photoinitiator, following the mechanism of step-growth addition and vinyl free radical polymerization. Chen et al. modified epoxidized soybean oil with multifunctional thiols or hydroxyl functionalized allyl compounds by Lewis acid catalyzed ring opening and prepared tack-free films with the glass transition temperature below 10 °C due to the flexible structure and thioether bonds formed by UV curing [19]. Kim et al. reported the synthesis of mercaptanized epoxidized soybean oil which was used to prepare the high biorenewable carbon content polymers with AESO by UV curing [20]. Mixed step-growth and chain-growth thiol−acrylate reactions of AESO and mercaptanized epoxidized soybean oil reduced the crosslinking density of the final polymers, resulting in higher elasticity compared to polymers formed only by chain-growth of AESO. Feng et al. reported thiol from gallic acid, which was used to obtain vegetable oil-based shape memory polymers [21]. The resulting polymers had comparable shape memory properties to the petroleum ones. Guzmán et al. synthesized trithiol from eugenol, which can be extracted from essential oils especially from clove oil, nutmeg, cinnamon, basil, and bay leaf [22]. The synthesized eugenol-based thiol led to more stable polymers obtained from cycloaliphatic resins than that obtained with petroleum-based thiol due to thermally labile ester groups in the latter. Şeker et al. synthesized dithiol derivative of isosorbide, which is considered to be plant-based monomer [23]. This isosorbide dithiol showed a better miscibility with tung oil compared to commercial thiol compounds and improved thermal properties and the surface hardness of the coatings due to rigid and thermally stable ring. Acosta Ortiz et al. prepared a hexathiol derivative of squalene (SQ6SH) through a thiol–ene reaction preceded by hydrolysis [24]. The flexible films were obtained when SQ6SH was used in UV curing reactions with petroleum-based unsaturated monomers. Although hexathiolated squalene has a flexible structure, the high functionality and the low molecular weight of each reactive unit could lead to rigid crosslinked material with the short distance between crosslinking points [22].

In this study, hexathiolated squalene was chosen for the synthesis of the crosslinked polymers as its starting compound squalene (SQ) is commercially available in large quantities by extraction from olives [25]. SQ is a natural compound (triterpene) synthesized in plants, animals, bacteria, algae, and fungi as a precursor for the synthesis of secondary metabolites such as sterols, hormones, or vitamins [25]. SQ (2,6,10,15,19,23-hexamethyl-6,6,10,14,18,20-tetracosahexane) was discovered in 1906 by Japanese researcher Tsujimoto Mitsumaru who separated the unsaponifiable fraction from the shark liver oil and named SQ under the denomination of the shark family *Squalidae* in Latin [26]. The use of SQ from marine animal oil has been limited by animal protection ant the presence of pollutants that cause cancer [27]. Therefore, the extraction of SQ from plants is a more preferred method. The highest concentration of SQ is in corn and amaranth (5942 mg/100 g), olive oil (564 mg/100 g), peanut (27.4 mg/100 g), rice, wheat germ, grape seed oil (14.1 mg/100 g), and soybean oil (9.9 mg/100 g) [25].

**Figure 1:**
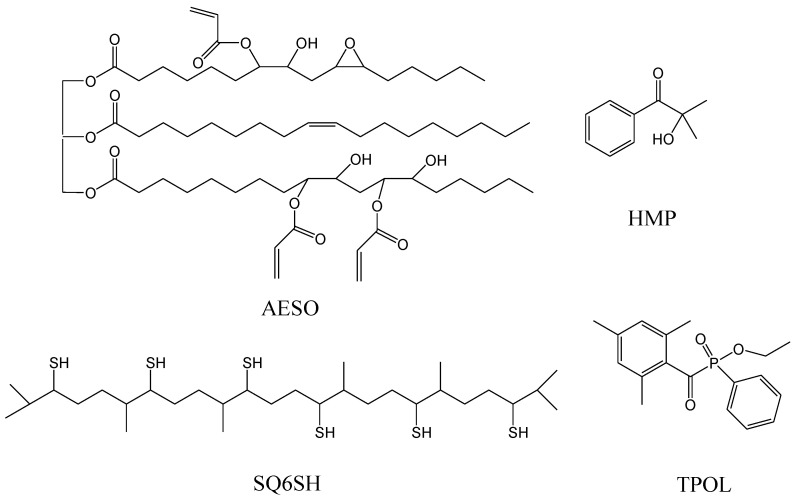
Chemical structures of acrylated epoxidized soybean oil (AESO), hexathiolated squalene (SQ6SH), 2-hydroxy-2-methylpropiophenone (HMP), and ethylphenyl (2,4,6-trimethylbenzoyl) phosphinate (TPOL).

Thiol–ene UV curing of two renewable monomers, AESO and hexathiolated squalene (SQ6SH) (Figure 1), was performed in this work. Two photoinitiators, 2-hydroxy-2-methylpropiophenone (HMP) and ethylphenyl (2,4,6-trimethylbenzoyl) phosphinate (TPOL) (Figure 1), were examined in thiol–ene reaction in different quantities to find out the influence of photoinitiators on the rate of UV curing. To the best of our knowledge, this is the first study of the UV curing process of AESO with SQ6SH and investigation of the synthesized polymer properties. Kinetics of UV curing process was monitored by the real-time photorheometry. Thermal properties and hardness of the obtained polymers were investigated. Furthermore, swelling values in polar and non-polar solvents were determined. Bio-based SQ6SH has previously been used in the thermal polymerization of AESO using 1-methylimidazole as a catalyst [28]. In the present study, photopolymerization was chosen, as it is a more efficient, environmentally friendly, energy saving, and economical method compared to thermal polymerization [29].

## 2. Materials and Methods

### 2.1. Materials

Acrylated epoxidized soybean oil (AESO, with 2.7 of acryloyl and 0.3 epoxide groups), squalene, thioacetic acid, 2,2-dimethoxy-2-phenylacetophenone, 2-hydroxy-2-methylpropiophenone (HMP), and ethylphenyl (2,4,6-trimethylbenzoyl) phosphinate (TPOL) were purchased from Sigma-Aldrich (Darmstadt, Germany). Inorganic salts were purchased from Scharlab (Barcelona, Spain). Methanol and chloroform were received from Carlo Erba (Barcelona, Spain). All materials were used as received.

### 2.2. Synthesis of Hexathiolated Squalene (SQ6SH)

SQ6SH was obtained by two-step procedure reported previously [24], which consisted of squalene thiol–ene UV curing resulting in hexathioacetate of squalene and hydrolysis reaction resulting in hexathiolated squalene (SQ6SH). SQ6SH was purificated by silica gel column chromatography and the product with the 70% yield was obtained. The description of ^1^H NMR and infra-red (IR) spectra can be seen in our previously published work [28].

### 2.3. Preparation of Crosslinked Polymers

Thiol–ene crosslinked polymers were obtained by photopolymerization of AESO and SQ6SH (acryl/SH groups 1:1) using different quantities (1–5 mol.%) of HMP or TPOL as photoinitiator under UV lamp (Helios Italquartz, model GR.E 500 W, Milan, Italy) at the distance of 15 cm with UV/Vis light at intensity of 310 mW/cm^2^ for 2 min at room temperature (20 °C). The polymer films of (0.51 ± 0.07) mm thickness were formed.

The resin and polymer codes consist of the photoinitiator concentration and the photoinitiator abbreviation. For example, 1HMP is a resin (or polymer) composed of AESO and SQ6SH (acryl/SH groups 1:1), and 1 mol.% of photoinitiator HMP.

### 2.4. Characterization

The ^1^H NMR spectra were registered with a Varian Gemini 400 spectrometer (Palo Alto, CA, USA) with CDCl_3_ as a solvent.

The reflection FT-IR spectra were recorded with a Perkin–Elmer (Llantrisant, UK) Spectrum BX II FT-IR spectrometer with the range of wavenumber of (650–4000) cm^−1^.

A MCR302 rheometer (Anton Paar, Graz, Austria) with the plate/plate accessory was used for photopolymerization kinetics of AESO and SQ6SH compositions (ratio of acrylate/SH groups 1:1) using 1–5 mol.% of photoinitiator (HMP or TPOL) (Table 1) at room temperature (25 °C). A OmniCure S2000 curing system (Lumen Dynamics Group Inc., Mississauga, Ontario, Canada) with UV/Vis radiation of 250–450 nm wavelength was used for the UV curing reactions through the glass plate. Shear mode with the frequency of 1 Hz and shear strain of 1% were used in all cases. Storage modulus G′, loss modulus G″, and complex viscosity η* values were taken after 300 s of composition irradiation by UV/Vis radiation. The G″ and G′ modulus crossover point was defined as gel point t_gel_. Resin shrinkage during the UV curing was determined as the difference between the gap before and after reaction. The mean values of three measurements of each resin were calculated.

The yield of insoluble fraction of polymers was determined using a Soxhlet extractor. A total of 0.2 g of each crosslinked polymer was extracted with the filter in a Soxhlet apparatus with acetone for 24 h and dried using vacuum at room temperature (20 °C). The yield of insoluble fraction was calculated as a difference between the polymer weight before and after extraction.

The swelling values were determined by measuring the weight of the samples swollen in acetone and toluene. The weighed samples (0.15 g) were placed into the vial of 10 mL with the solvent at room temperature (20 °C). The samples were weighed every 15 min after cleaning the solvent for the surface.

Biorenewable carbon (BRC) was calculated by evaluating the ratio between bio sourced carbon and fossil carbon.

Differential scanning calorimetry (DSC) and a DSC 8500 apparatus (Perkin–Elmer, Llantrisant, UK) were used to determine the glass transition temperature (T_g_). The measurements were performed with a 10 °C·min^−1^ heating-cooling-heating rate and nitrogen flow rate 50 mL·min^−1^.

The thermal stability of polymers was determined by thermogravimetric analysis (TGA) and a Perkin–Elmer TGA 4000 apparatus with a heating rate of 20 °C·min^−1^ and nitrogen flow rate 100 mL·min^−1^.

Shore A hardness of polymers was determined using Sauter durometer HBA 100-0 (Kern & Sohn GmbH, Balingen, Germany). Samples of 12 mm thickness were used for the measurements. The mean values of three measurements were calculated.

## 3. Results and Discussion

### 3.1. Photopolymerization Kinetics

**Figure 2:**
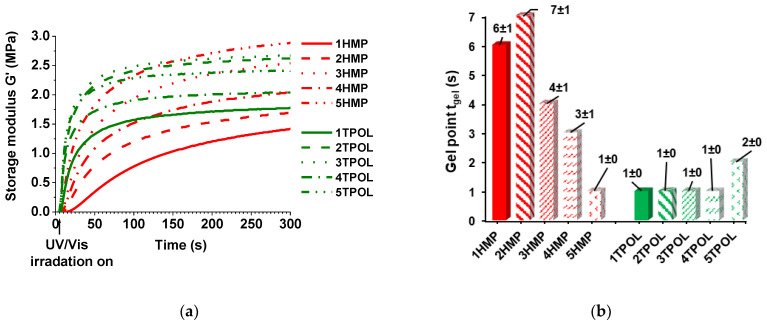
(**a**) Curves of storage modulus G′ versus irradiation time of the compositions with different photoinitiators, (**b**) Gel point of AESO thiol–ene resins.

The real-time photorheometry data of AESO-SQ6SH thiol–ene resins are summarized in Table 1. The highest values of storage modulus G′, loss modulus G″, and complex viscosity η* were obtained when 5 mol.% of HMP and 3 mol.% of TPOL were used. G′ modulus values (2.95 MPa and 2.67 MPa, respectively) were similar to those of AESO polymers with petroleum-based aromatic dithiols tested by the same real-time photorheometry method (2–9 MPa) [13]. As an example, the typical curves of storage modulus G′ are presented in Figure 2a. G′ increased very fast when irradiation of the resins started. This shows the quick chain size growth and the network formation in the initial part of photocrosslinking. With the increase of the photoinitiator concentration, G′ modulus changed unevenly due to formation of higher concentration of active species and competition with double bonds during the initiation stage. G″ and η* values increased in the same order as G′ values. G′ of resins with TPOL increased very fast during the first 10 s and continued to increase slowly with time due to the gel aging and settled down into a steady-state (plateau) indicating the end of the gelation process. However, G′ of resins with HMP increased more slowly due to the lower activity of HMP compared to TPOL. t_gel_ was reduced when TPOL was used as photoinitiator although obtained G′, G″, and η* values were similar. The concentration of photoinitiator had influence on the gel point t_gel_ (Figure 2b). The lowest t_gel_ value was determined when 1–4 mol.% of TPOL was used. However, using higher 5 mol.% concentration of TPOL, the t_gel_ value increased due to generated higher concentration of free radicals at the surface. This led to reduced rate of polymerization in the deeper layers due to blocking the sufficient energy from penetrating [30]. The opposite results were obtained using photoinitiator HMP in the resins. The highest G′, G″, and η* values and the lowest t_gel_ value were determined when 5 mol. % of HMP was used probably due to the occurrence of terminated reactions of macroradicals with free radicals [31].

It is known that the shrinkage of the resins is influenced by the change of inter-molecular distances between covalent bond and Van der Waals force during the polymerization [32]. However, the shrinkage of TPOL resins was higher compared to HMP resins due to faster reaction as the t_gel_ values were lower compared to HMP resins. Moreover, the AESO and SQ6SH resins had lower shrinkage (3.0–6.5%) compared to those of AESO resins with various plant-derived acrylates (8.0–13.3%) [8]. This can be explained by the higher viscosity of AESO and SQ6SH resins.

### 3.2. Characterization of the Crosslinked Polymer Structure

The UV-cured thiol–ene polymers obtained from AESO and SQ6SH were insoluble in common organic solvents. To confirm their crosslinked structure, the Soxhlet extraction was performed (Table 2). The values of the yield of insoluble fraction were in the range of 68–98%. The highest yield of insoluble fraction was obtained when 3 mol.% of HMP and TPOL were used (90% and 98%, respectively) due to the formation of optimal concentration of active species which could initiate the reaction. This could be explained by the theory that polymerization of the resin rise with the increased concentration of photoinitiator until reaches optimal concentration and then declines [33]. By increasing amount of photoinitiator HMP or TPOL until 3 mol.% in the resin, the yield of insoluble fraction of polymers increased. After optimal concentration of 3 mol. %, the yield of insoluble fraction decreased. More free radicals were formed and terminated reactions of macroradicals with free radicals occurred. The yield of insoluble fraction of above 90% was observed for the polymers when TPOL was due to the higher activity of TPOL and its ability to generate free radical fast which enabled fast crosslinking and high yield of insoluble fraction.

**Figure 3:**
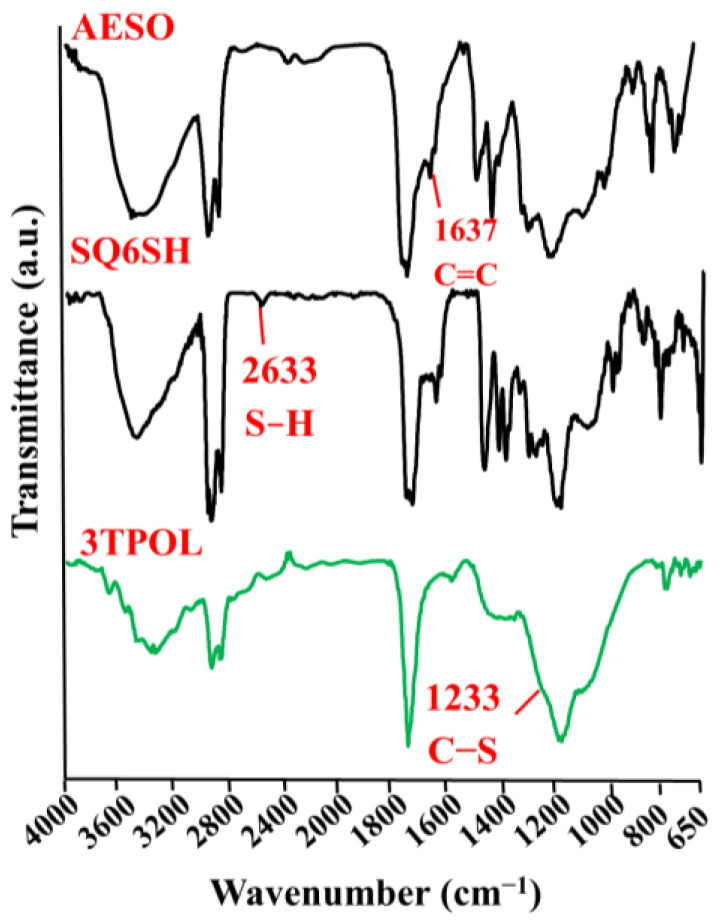
FT-IR spectra of AESO, SQ6SH, and 3TPOL.

Crosslinked polymers were characterized by FT-IR spectroscopy, which spectra showed the characteristic absorption signals corresponding to their chemical structure. As an example, FT-IR spectra of monomers AESO and SQ6SH, and polymer 3TPOL are presented in Figure 3. The S–H group signal present at 2633 cm^−1^ in the spectrum of SQ6SH and the signal of C=C at 1637 cm^−1^ which was present in the spectrum of AESO disappeared in the FT-IR spectra of all polymers. The C–S group signal at 1233 cm^−1^ appeared in the FT-IR spectra of the crosslinked polymers.

**Figure 4:**
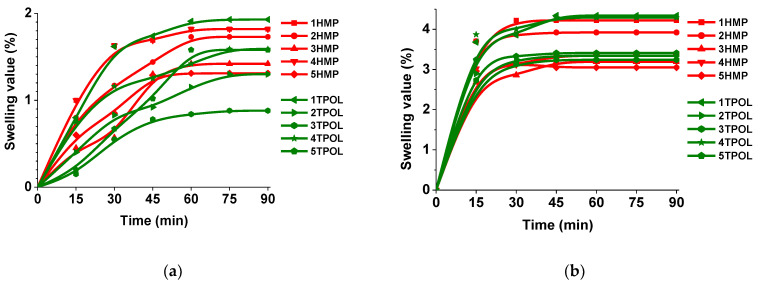
(**a**) Swelling value versus time curves of the polymers in acetone, (**b**) Swelling value versus time curves of the polymers in toluene.

Swelling capacity is related to the crosslinking density of polymers, also swelling is an important parameter of polymer for their potential application in various areas where swelling is essential. Swelling value versus time curves are presented in Figure 4. The swelling equilibrium was reached after 60 min in acetone and after (30–45) min in toluene by all polymers. Swelling values of polymers in acetone and toluene were very low due to the high crosslinking density and short distances between crosslinking points of the polymer network. Therefore, these polymers could be used where low swelling is significant. The highest swelling degree observed after 90 min in acetone was 1.93% and that in toluene was 4.34%. They were much lower compared to the swelling values of the thermally cured AESO and SQ6SH polymer (61–64%) [28]. This suggests that the density of crosslinks of photopolymers is much higher. Correlation between swelling value and the crosslinking density (related to the G′ values) was found. Polymers with higher crosslinking density were less swollen. This observation can be explained by the formation of the shorter chains between the crosslinking points of the network.

The synthesis of high biorenewable content materials that match or even surpass the properties of petroleum-based materials with competitive cost is important to replace petroleum-based materials. High BRC materials can be obtained by designing and preparing the resins from renewable raw materials [34]. BRC was found to be 88–89% for all AESO and SQ6SH polymers synthesized in this work and was higher compared to that of AESO coatings with petroleum-based monomers (~50%) [34] and AESO polymers with plant-derived acrylates (75–82%) [8].

### 3.3. Thermal Properties of the Polymers

**Figure 5:**
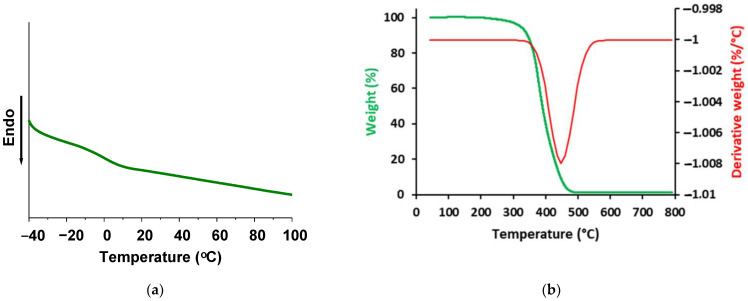
DSC curve (**a**) and thermogravimetric curves (**b**) of polymer 3TPOL.

Thermal characteristics of polymers investigated by DSC and TGA are summarized in Table 2. Glass transition temperatures (T_g_) of polymers synthesized using photoinitiator TPOL were below 0 °C but higher than those of polymers synthesized with photoinitiator HMP (from −20 °C to −6 °C) and were similar to those of dual cured AESO polymers synthesized with petroleum-based thiols (from −4 °C to 4 °C) [17] and thermally cured AESO and SQ6SH polymer (−14 °C) [28]. Such low T_g_ values of AESO and SQ6SH polymers could be caused by the presence of the flexible thioether linkages [35]. The highest T_g_ was obtained (0 °C) when 3 mol.% of TPOL was used due to the high yield of insoluble fraction and crosslinking density (Figure 5a). The same behavior was noticed with the temperature at the weight loss of 5% and 10% (T_dec.−5%_, T_dec.−10%_). This could be explained by the higher yield of insoluble fraction and crosslinking density of the crosslinked polymers. Polymer 3TPOL with the highest yield of insoluble fraction (98%) exhibited the highest T_dec.−5%_, and T_dec.−10%_ values (322 °C, and 344 °C, respectively). These values were similar to those of AESO polymer with petroleum-based aromatic dithiol (342 °C) [13]. T_dec.−5%_ of all polymers (300–322 °C) was higher compared to that of eugenol-based thiol–ene polymer where SQ6SH was used as a crosslinker (252 °C) [36] probably due not fully cured eugenol-based polymer. The values of temperature of the maximum rate of one-step degradation (T_max_) (446–456 °C) were independent from crosslinking density. Thermal degradation of obtained AESO and SQ6SH polymers proceeded in one degradation step which shows similar bond thermal stability of crosslinked networks and is related to the high crosslinking density [37] (Figure 5b). The derivative curve shows that degradation begins at 350 °C and a maximum degradation rate is above 445 °C. The char residue of all polymers after degradation was very low ~1% which shows non-aromatic structure of polymers and was similar to the char residue of dual cured AESO polymers with petroleum-based thiols [17] and thermally cured AESO and SQ6SH polymer [28]. The char residue values of polymers differ slightly and were independent from crosslinking density.

### 3.4. Hardness of the Polymers

**Figure 6:**
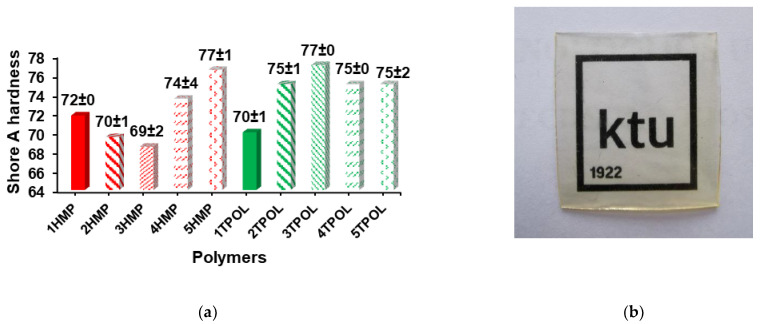
(**a**) Shore A hardness of AESO thiol–ene polymers, (**b**) Photograph of polymer 3TPOL.

The Shore A hardness data of polymers are collected in Figure 6a. Shore A hardness values of polymers were in the range of 69–77 and depended on the polymer crosslinking density and the yield of insoluble fraction. Polymers 5HMP and 3TPOL with high crosslinking density and the yield of insoluble fraction showed the highest Shore A hardness (77) which was higher compared to the thermally cured AESO and SQ6SH polymer (62) [17] and comparable to Shore A hardness of the tung-oil-based polymers synthesized with petroleum-based thiols (65–73) [23].

The photograph of the polymer 3TPOL film is shown in Figure 6b. The obtained polymer film was transparent, yellowish, and smooth.

## 4. Conclusions

High biorenewable carbon content and highly photocrosslinked polymers have been synthesized from functionalized soybean oil and squalene by thiol–ene UV curing. Hexathiolated squalene was prepared from squalene as a starting bio-based material and was used as a crosslinking agent in the thiol–ene UV curing of acrylated epoxidized soybean oil using two different photoinitiators, 2-hydroxy-2-methylpropiophenone and ethylphenyl (2,4,6-trimethylbenzoyl) phosphinate, in different quantities. The rate of photopolymerization was the highest when 3 mol.% of ethylphenyl (2,4,6-trimethylbenzoyl) phosphinate was used. Polymers had the higher storage modulus (1.78–2.67 MPa), glass transition temperature (−2–0 °C), Shore A hardness value (70–77), and lower swelling value (0.88–1.93% in acetone and 3.24–4.34% in toluene) due to the higher crosslinking density and the yield of insoluble fraction when ethylphenyl (2,4,6-trimethylbenzoyl) phosphinate was used in the resins. The rheological, thermal properties and hardness of polymers synthesized from functionalized soybean oil and squalene by UV curing were comparable with those of acrylated epoxidized soybean oil polymers with petroleum-based thiols and thermally cured bio-based acrylated epoxidized soybean oil and hexathiolated squalene polymers.

## Figures and Tables

**Table 1:** Rheological characteristics and shrinkage of AESO thiol–ene resins.

Resin	Storage Modulus G′ (MPa)	Loss Modulus G″ (MPa)	Complex Viscosity η* (MPa·s)	Shrinkage (%)
1HMP	1.41 ± 0.02	0.13 ± 0.02	228.0 ± 1.0	3.0 ± 0.0
2HMP	1.71 ± 0.01	0.23 ± 0.01	274.0 ± 0.5	5.0 ± 0.0
3HMP	2.54 ± 0.02	0.41 ± 0.01	409.5 ± 1.5	3.0 ± 0.0
4HMP	2.03 ± 0.02	0.32 ± 0.01	326.5 ± 3.5	3.5 ± 0.5
5HMP	2.95 ± 0.02	0.48 ± 0.01	475.0 ± 2.0	2.5 ± 0.5
1TPOL	1.78 ± 0.02	0.19 ± 0.00	285.5 ± 1.5	3.5 ± 0.5
2TPOL	2.61 ± 0.02	0.36 ± 0.01	419.5 ± 2.5	5.0 ± 0.0
3TPOL	2.67 ± 0.00	0.38 ± 0.00	428.5 ± 1.5	4.0 ± 0.0
4TPOL	2.05 ± 0.00	0.27 ± 0.00	330.0 ± 0.5	6.5 ± 0.5
5TPOL	2.41 ± 0.01	0.32 ± 0.01	388.0 ± 1.0	6.0 ± 0.0

**Table 2:** Thermal characteristics and yield of insoluble fraction of the crosslinked polymers.

Polymer	Yield of Insoluble Fraction ^a^ (%)	T_g_ ^b^ (°C)	T_dec.−5%_^c^ (°C)	T_dec.−10%_^d^ (°C)	T_max_ ^e^ (°C)	Char Residue ^f^ (%)
1HMP	68	−20	305	333	450	1.1
2HMP	85	−6	309	333	447	1.2
3HMP	90	−7	306	330	452	0.9
4HMP	88	−9	300	331	456	0.5
5HMP	87	−11	303	331	448	0.9
1TPOL	93	−1	314	340	447	1.2
2TPOL	97	−1	316	342	449	1.1
3TPOL	98	0	322	344	448	1.2
4TPOL	97	−2	313	340	449	0.9
5TPOL	96	−2	305	334	446	0.9

^a^ After 24 h Soxhlet extraction with acetone; ^b^ Glass transition temperature estimated by DSC; ^c^ Temperature at the weight loss of 5% obtained from TGA curve; ^d^ Temperature at the weight loss of 10% obtained from TGA curve; ^e^ Temperature of the maximum rate of one-step degradation obtained from derivative weight loss curve; ^f^ After thermal degradation in N_2_ atmosphere.

## Data Availability

The data presented in this study are available on request from the corresponding author.
